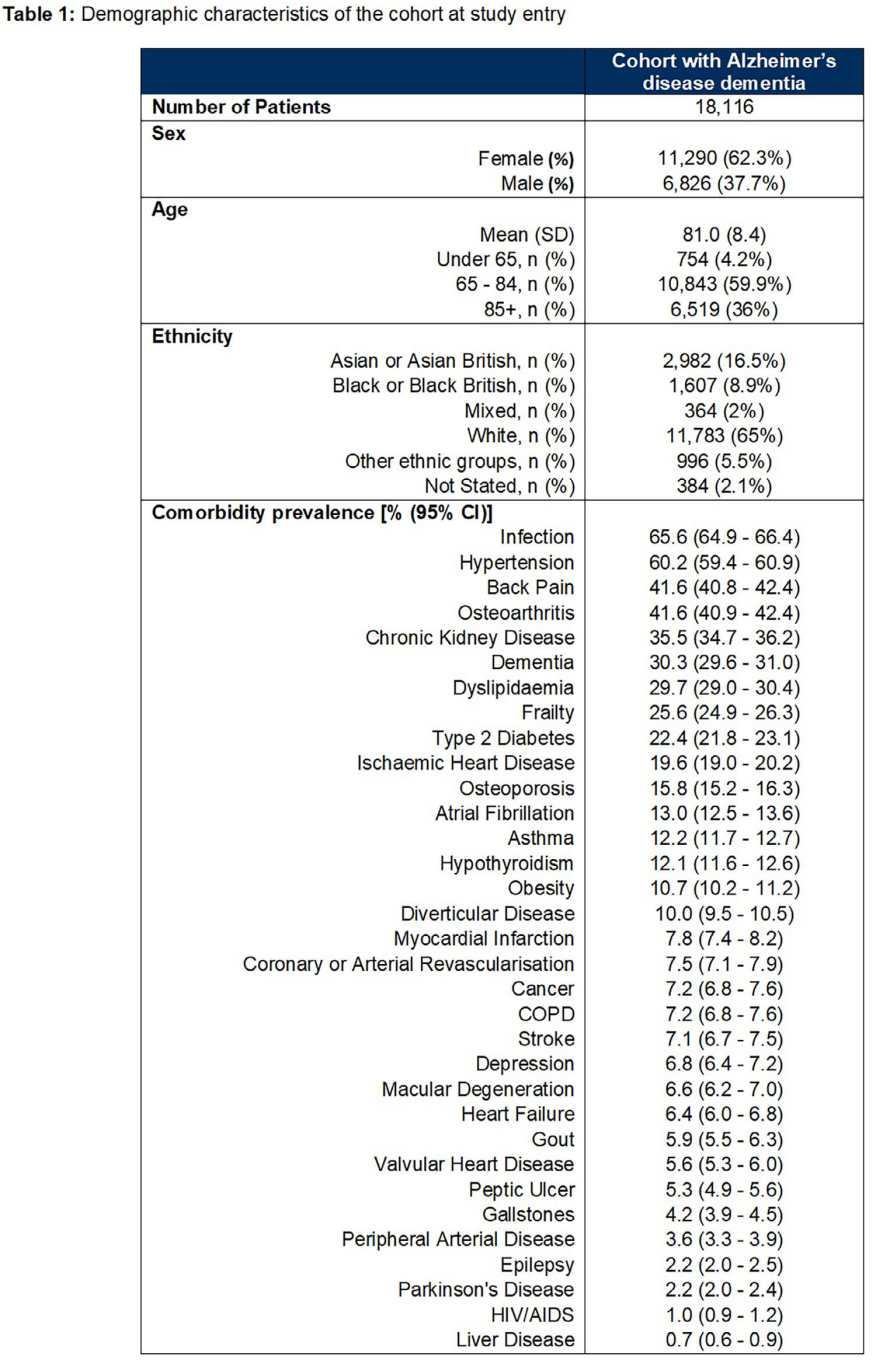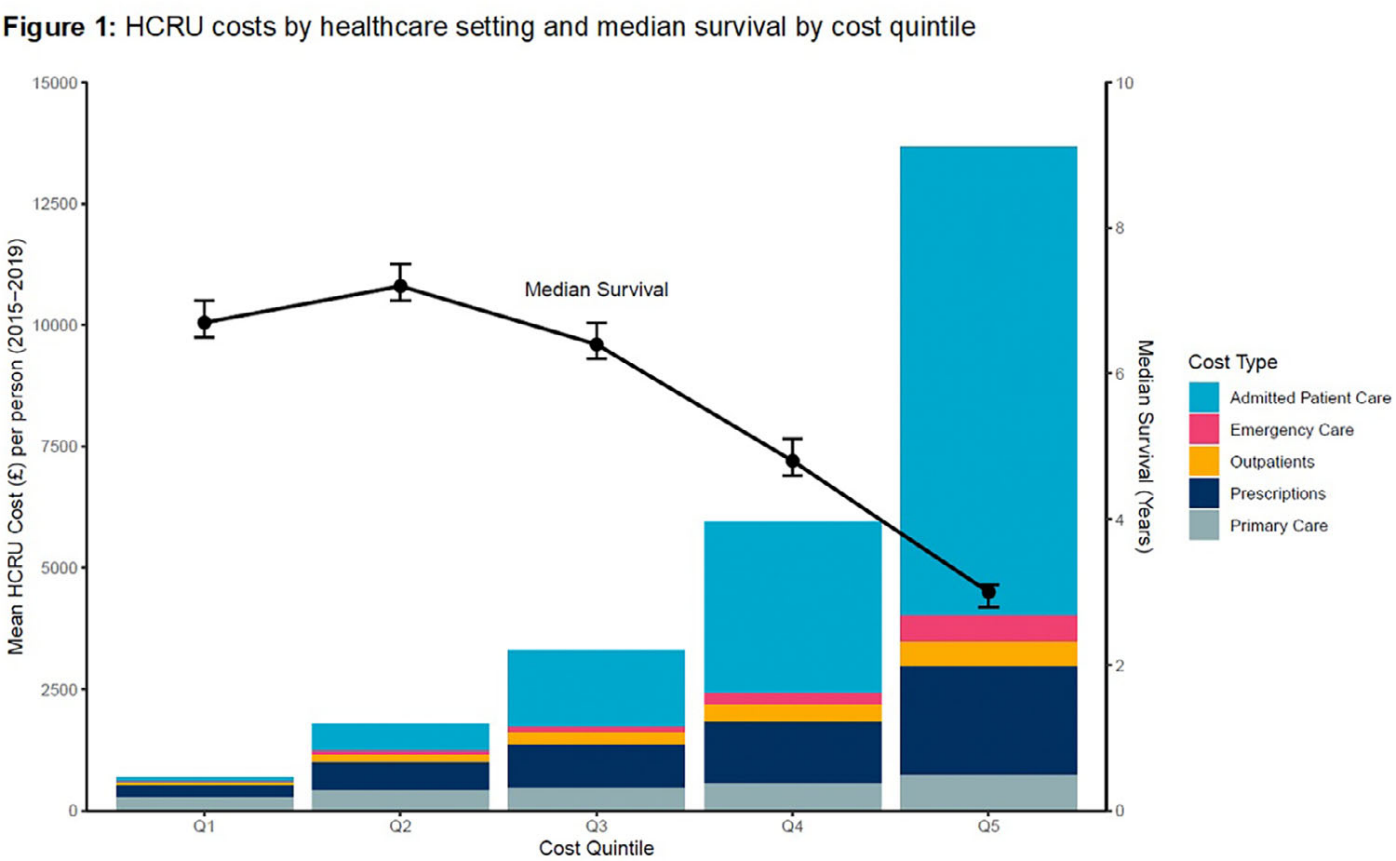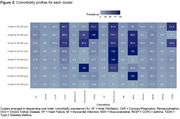# Characterising subpopulations of patients with Alzheimer’s disease dementia who have high healthcare utilisation and costs

**DOI:** 10.1002/alz.087465

**Published:** 2025-01-09

**Authors:** Sophie Edwards, Dominic Trepel, Craig W Ritchie, Julie Hviid Hahn‐Pedersen, Mei Sum Chan, Benjamin D Bray, Alice Clark, Christian Ahmad Wichmann, Marc Evans

**Affiliations:** ^1^ Barts Health NHS Trust, London United Kingdom; ^2^ Trinity College Dublin, Dublin Ireland; ^3^ Scottish Brain Sciences, Edinburgh United Kingdom; ^4^ University of Edinburgh, Edinburgh United Kingdom; ^5^ Novo Nordisk A/S, Søborg Denmark; ^6^ Lane Clark & Peacock LLP, London United Kingdom; ^7^ University Hospital Llandough, Llandough United Kingdom

## Abstract

**Background:**

How and why healthcare utilisation and costs vary between patients with Alzheimer’s disease dementia (ADD) is not well understood but is important in ensuring that efforts to improve the diagnosis and treatment of ADD are prioritised effectively. We aimed to investigate variation in healthcare resource utilisation (HCRU) among patients with ADD in England and identify the clinical and demographic factors which characterise subpopulations with the highest HCRU.

**Method:**

This was a retrospective cohort study using Discover, a linked electronic health record database of 2.8 million residents in London, England. We identified individuals with ADD using diagnostic codes and estimated HCRU and total healthcare costs over up to 9 years of follow up (2010‐2019). Individuals included in the 2019 cross‐section were stratified into cost quintiles, and K‐medoids clustering was used to identify subpopulations with high costs.

**Result:**

We identified 18,116 individuals with ADD between 2010 and 2019 (Table 1). Costs in the highest HCRU cost quintile in 2019 accounted for 56% of all healthcare costs in the ADD population, were approximately 20 times higher than the lowest cost quintile (£13,665 vs. £678 per patient per year [ppy]) and were largely attributed to hospital admissions (Figure 1). Individuals in the higher cost populations tended to be older, male, have a higher comorbidity burden and a lower median survival than those in the lower cost populations. The three groups with the highest costs identified by the cluster analysis had substantial comorbidity burden, with more than 65% of individuals having four or more comorbidities (Figure 2). The most common comorbidities were cardiometabolic diseases, chronic kidney disease, and frailty. The costs in the highest cost cluster were more than double the cluster with lowest costs (£6,355 vs. £3,160 ppy).

**Conclusion:**

Healthcare utilisation and costs in individuals with ADD vary widely, with a large proportion of costs being attributable to a minority of patients with multiple comorbidities, particularly cardiometabolic diseases and frailty. Health systems should focus on patients with clinical profiles associated with high costs in efforts to improve the timely diagnosis and treatment of ADD and mitigate the financial costs of ADD on patients, health systems, and society.